# Overexpression of DNA Polymerase Zeta Reduces the Mitochondrial Mutability Caused by Pathological Mutations in DNA Polymerase Gamma in Yeast

**DOI:** 10.1371/journal.pone.0034322

**Published:** 2012-03-28

**Authors:** Enrico Baruffini, Fausta Serafini, Iliana Ferrero, Tiziana Lodi

**Affiliations:** Department of Genetics, Biology of Microorganisms, Anthropology, Evolution, University of Parma, Parma, Italy; University of Medicine and Dentistry of New Jersey, United States of America

## Abstract

In yeast, DNA polymerase zeta (Rev3 and Rev7) and Rev1, involved in the error-prone translesion synthesis during replication of nuclear DNA, localize also in mitochondria. We show that overexpression of Rev3 reduced the mtDNA extended mutability caused by a subclass of pathological mutations in Mip1, the yeast mitochondrial DNA polymerase orthologous to human Pol gamma. This beneficial effect was synergistic with the effect achieved by increasing the dNTPs pools. Since overexpression of Rev3 is detrimental for nuclear DNA mutability, we constructed a mutant Rev3 isoform unable to migrate into the nucleus: its overexpression reduced mtDNA mutability without increasing the nuclear one.

## Introduction

In yeast, DNA polymerase zeta (Pol zeta) and Rev1 are involved in the error-prone translesion synthesis (TLS) during replication of nuclear DNA: mutations in any of these polymerases lead to a decrease of mutagenesis in nuclear genome either in normal conditions or in conditions known to increase DNA damage such as UV treatment [1]. The yeast Pol zeta is composed by a catalytic subunit encoded by *REV3* and an accessory subunit encoded by *REV7*. Biochemically, Pol zeta is a poorly processive enzyme [2], lacks of a 3′-5′ exonucleolytic proofreading activity [3] and is able to extend terminally mismatched primers [4]–[6]. From these properties derives its propensity to generate mutations. On the contrary, Pol zeta is very inefficient in inserting nucleotides opposite lesions *in vitro*, suggesting that a distinct polymerase should act in this first step and that Pol zeta can efficiently extend from primer-terminal base pairs containing mismatches or lesions [6].

Rev1 encodes for a deoxycytidyl transferase that preferentially incorporates C opposite to an abasic site [7]. Also, Rev1 interacts *in vitro* with Pol zeta through a Rev3-Rev1 binding and this interaction strongly stimulates the activity of Pol zeta and, probably, contributes to its targeting to the replication fork [8].

Recently, it was found that Pol zeta and Rev1 localize in mitochondria and that they participate in the mitochondrial DNA mutagenesis, since disruption of *REV3*, *REV7* or *REV1* resulted in a strongly decreased frequency of −1 frameshift mutations in mtDNA [9]. On the contrary, it was shown that deletion of *REV3*, *REV7* or *REV1* resulted in increased frequencies of both spontaneous and UV-induced mitochondrial erythromycin resistance, due to base substitutions [10]. On the basis of their results, these authors speculated that, in absence of the TLS by Rev1 and/or Pol zeta, the mitochondrial DNA polymerase gamma (Pol gamma), the mitochondrial replicase, could create mutations in efforts to bypass lesions that block the replication fork [10]. As a consequence, Pol zeta and Rev1, which in the nucleus are responsible with other DNA polymerases for the error-prone bypass of DNA lesions, in the mitochondrial compartment function in a less mutagenic pathway [10].

Pol gamma is a protein conserved in fungi and animals. Structurally, human Pol gamma is a heterotrimer composed by one catalytic subunit, encoded by *POLG*, and two accessory subunits, encoded by *POLG2* [11]. The catalytic subunit contains an N-terminal 3′-5′ exonuclease domain (residues 170–440), a C-terminal polymerase domain (residues 440–475 and 785–1239) and a spacer region encompassing residues 475–785 [12]. To date, more than 150 pathological mutations in *POLG* have been identified in severe mitochondrial disorders (http://tools.niehs.nih.gov/polg/) and, among them, a few mutations have been recently described in stavudine induced toxicity [13], [14], in valproate induced hepatotoxicity [15], [16] and in breast tumorigenesis [17]. This places *POLG* as a major locus for mitochondrial diseases and disorders.

In yeast the DNA polymerase gamma, encoded by *MIP1* gene, is composed by a sole catalytic subunit [18]. Thanks to the similarity between human Pol gamma and Mip1 (approximately 43%), yeast has been used to validate the role of human putative pathological mutations, to understand the biochemical consequences associated to these mutations, to study the pharmacogenetics of drugs such as valproate and stavudine, and to find mechanisms able to rescue the detrimental effects of Mip1 mutations [16], [19]–[28]. Since a therapy has not been yet developed for mitochondrial diseases caused by *POLG* mutations, a major goal of the research involving yeast/animal models is to find new strategies to rescue the pathological phenotypes associated to these mutations.

Yeast is a suitable model organism to study the effects of nuclear mutations affecting mtDNA stability, thanks to its ability to survive in absence of a functional mitochondrial genome and to the possibility to easily analyze mtDNA mutability. Yeast cells containing deletions-carrying mtDNA, called *rho-*, or cells which have completely lost mtDNA, called *rho^0^*, are respiratory deficient and produce colony of small size, called *petite*. *rho^−^* cells, which arise more frequently than *rho^0^* cells, retain only small fragments of wt mtDNA, from 30% to less than 1% (reviewed in [29]). Although the mechanisms thanks to which *rho^−^* mutations arise are not fully know, analysis of the regions flanking the deleted regions suggest that mtDNA of some of these *rho^−^* cells derives from recombination between direct repeats flanking the deleted region with consequent excision [29]. In addition, deletions do not occur randomly at all. As a matter of fact, some fragments, encompassing *COB*, *COX2* and *COX3* genes, are most frequently retained [29]. These large deletions make mtDNA irreversibly unfunctional. In *S. cerevisiae*, *petite* mutants arise spontaneously with high frequency (approximately 10^−2^) [29]. The frequency of *petite* mutants is a measure of mtDNA extended mutability and is an index of mtDNA instability. Extended mutability is indeed a series of large deletions (generally >70%) that arise spontaneously, and partially randomly, in the mtDNA, without involvement of point mutations. Mutations in a large group of nuclear genes, encoding for proteins directly or indirectly involved in mtDNA replication, recombination, stability and maintenance (reviewed in [30]) produce increase of *petite* mutability. mtDNA point mutations can be also easily measured as frequency of spontaneous mutants resistant to erythromycin (Ery^R^ mutants), an antibiotic that inhibits mitochondrial but not cytoplasmic translation. In fact, resistance to erythromycin is acquired through specific transversions or transitions in the mitochondrial gene encoding the 21 S rRNA, in particular at 1950, 1951, 1952 and 3993 positions [31]–[34]. Ery^R^ mutants arise spontaneously approximately at a frequency of 10^−7^–10^−8^. Ery^R^ mutability is influenced by replication fidelity of DNA polymerase gamma as well as by the activity of some enzymes involved in mtDNA repair and/or recombination [34]–[37].

In an attempt to investigate about genetic conditions that might reduce the damages caused by mutations in POLG, we analyzed the effect of deletion or overexpression of TLS polymerases that, together with DNA polymerase gamma, are present in mitochondria, Pol zeta (Rev3–Rev7) and Rev1. Genetic tests based on gene overexpression are widely used in yeast to assess a role of a gene function in a biological process. Several researches are reported where one or more proteins were overexpressed with this aim, among which a study showing that overexpression of Rev3 led to increasing UV-induced mutagenesis [38]. In addition, gene overexpression is commonly used to rescue the phenotypic defects caused by mutations in another gene which is functionally associated. For example, in the case of *MIP1*, overexpression of *RNR1*, encoding the large subunit of the ribonucleotide reductase, lead to the discovery that increased levels of dNTP pools reduced the extended mutability caused by mutations in Mip1 [19], [26], [39]. This finding has had impacted also in studies on human DNA polymerase gamma pathological mutations [26], where, starting from analysis in yeast, it has been shown that human DNA polymerase gamma harboring the pathological mutation H932Y has a ∼200-fold reduced affinity to the incoming dNTPs.

Here we found that increased expression of Pol zeta resulted in a significant reduction of extended mutability caused by mutations mapping in different domains of *MIP1* and that overexpression of both Pol zeta and Rev1 led to a reduction in mtDNA point mutability.

## Materials and Methods

### Yeast strains and media

The yeast strains used in this work are DWM-5A (*Matα ade2-1 leu2-3,112 ura3-1 trp1-1 his3-11,15 can1-100 mip1::Kan^R^*), DYY-4C (*Mata ade2-1 leu2-3,112 ura3-1 trp1-1 his3-11,15 can1-100 mip1::Kan^R^ sml1::HIS3*) [19], W303-1A CAN^S^ (*Matα ade2-1 leu2-3,112 ura3-1 trp1-1 his3-11,15*) (a kind gift from Antoni Barrientos, University of Miami Miller School of Medicine, Miami, FL, USA), W303-1B (*Mata ade2-1 leu2-3,112 ura3-1 trp1-1 his3-11,15 can1-100*) [40], BY4742 *rev1Δ* (*Matα, his3Δ1, leu2Δ0, lys2Δ0, ura3Δ0 rev1::Kan^R^*), BY4742 *rev7Δ* (*Matα, his3Δ1, leu2Δ0, lys2Δ0, ura3Δ0 rev7::Kan^R^*) (Euroscarf collection), DWM-5Ar3 (*Matα ade2-1 leu2-3,112 ura3-1 trp1-1 his3-11,15 can1-100 mip1::Kan^R^ rev3::Hph^R^*), DWM-9A (*Matα ade2-1 leu2-3,112 ura3-1 trp1-1 his3-11,15 can1-100 mip1::Hph^R^*) [41], DWM-9Ar1 (*Matα ade2-1 leu2-3,112 ura3-1 trp1-1 his3-11,15 can1-100 mip1::Hph^R^ rev1::Kan^R^*), DWM-9Ar7 (*Matα ade2-1 leu2-3,112 ura3-1 trp1-1 his3-11,15 can1-100 mip1::Hph^R^ rev7::Kan^R^*).

Synthetic defined medium (SD) contained 6.9 g/l yeast nitrogen base without amino acids (Formedium), synthetic complete medium (SC) was SD supplemented with 1 g/l drop out mix [42]. YP medium contained 1% yeast extract (Formedium) and 2% peptone (Formedium), and YPA medium was YP supplemented with 40 mg/ml adenine base (Formedium). Carbon sources were added as indicated in the text. YPAEG-Ery contained 1% yeast extract (Difco), 2% Bacto-peptone (Difco), 40 mg/l adenine base, 25 mM potassium phosphate pH 6.5, 3% ethanol, 3% glycerol and 3 g/l erythromycin (Sigma). CAN plates was SD medium supplemented with 0.006% L-canavanine sulfate (Sigma), amino acids and bases necessary to complement the auxotrophy and 2% glucose.

### Plasmids construction

The DNA constructions produced in this work were obtained using plasmids pFL38, which is centromeric and brings the *URA3* marker, pFL39 (centromeric, *TRP1* marker) [43], YEplac195 (multicopy, *URA3* marker), YEplac181 (multicopy, *LEU2* marker) [44], pUG35 (centromeric, *URA3* marker, for EGFP fusion) (http://mips.helmholtz-muenchen.de/proj/yeast/info/tools/hegemann/gfp.html), pTL85 (multicopy, *Kan*MX4 marker, for EGFP fusion) [41] and pAG32, an integrative plasmid used as template for gene disruption with the *Hph*MX4 cassette [45].

Plasmids pFL38-*MIP1* and pFL39 harboring wt or mutant *mip1* alleles have been previously described [19]. YEplac195-*REV3* was obtained by PCR-amplification of *REV3*, including the upstream and the downstream regions, from genomic DNA of strain DWM-5A with primers REV3-Fw (gcgcgggtaccggatccaagaatccctgtgg) and REV3-Rv (gcgccgaattccttagaggatacgaagattcc), digestion with *Kpn*I and *Eco*RI of both the PCR fragment and YEplac195 and ligation. YEplac195-*REV7* was obtained by PCR-amplification of *REV7* from genomic DNA of strain DWM-5A with primers REV7-Fw (gcgccgtcgacgatgaatgggaaaagaagtgc) and REV7-Rv (gcgcgggtacccatggaaccagacagaagacc), digestion of both the PCR fragment and YEplac195 with *Sal*I and *Kpn*I and ligation. YEplac195-*REV3/REV7* was obtained by subcloning *REV7* from YEplac195-*REV7* to YEplac195-*REV3* digested with *Sal*I and *Kpn*I. YEplac181-*REV1* was obtained by PCR amplification of *REV1* from genomic DNA of strain BY4742 with primers REV1-Fw (cggcgggtcgacgtgaagtgatcatgcacatcg) and REV1-Rv (cggccggtacccttacgcatttctactgttgg), digestion of both the PCR fragment and YEplac181 with *Sal*I and *Kpn*I and ligation. pUG35-REV3 was obtained by PCR amplification of *REV3* ORF from genomic DNA of strain DWM-5A with primers REV3GFPFw (ccgccgaattcatgtcgagggagtcgaacgacac) and REV3GFPRv (ccgccgtcgacccaatcatttagagatattaatgc), digestion of both the PCR fragment and pUG35 with *Eco*RI and *Sal*I and ligation in order to clone *REV3* in frame with the EGFP gene. pTL85-REV3 was obtained by subcloning *REV3* from pUG35-*REV3* to pTL85 digested with *Xba*I and *Sal*I. The *REV3* DNA region corresponding to the amino acid stretch 1 to 534 was cloned in frame with the EGFP gene of pUG35 by amplification of the fragment from genomic DNA of strain BY4742 with primers REV3GFPFw and R3GFPRvS (ggcccgtcgaccagaatatcttgataaccg) and cloning it between the *Eco*RI and *Sal*I sites. All the cloned fragments were sequenced to check for the absence of mutations.

### *REV3* mutagenesis

505–508 RKRKK stretch of Rev3 was mutagenized to AAAAA through the overlap extension technique [46]. In the first set of PCR reactions, the *REV3* fragment encoding for the 1–534 Rev3 stretch was amplified with i) the primer REV3GFPFw and the mutagenic primer REV3NONSRV (cgtaatcccgcagccataaccGCcGCtGCgGCcGCagacagcaacgatgttttattagc), and ii) the mutagenic primer REV3NONSFW, complementary to REV3NONSRV, and the R3GFPRVS primer. The final mutagenized product was obtained by using the overlapping PCR fragments as template and REV3NONSFW and R3GFPRVS as external primers. The product was then digested with *Eco*RI and *Sal*I and cloned in *Eco*RI-*Sal*I digested pUG35.

To mutagenize YEPlac195-*REV3*, a fragment of *REV3* was amplified with the primer REV3MUTFW (cattgagacaccaaagatgtgc) and with the mutagenic primer REV3NONSRV, and with the mutagenic primer REV3NONSFW and the primer REV3MUTRV (cagaatggaaatctattcctatc). The final product was obtained as above and was cloned in *Ngo*MIV-*Sac*I digested YEplac195-*REV3*. The entire cloned fragment was sequenced to confirm the presence of the mutations.

### Disruption of *REV3*, *REV7* and *REV1*

*REV3* was disrupted in strain DWM-5A by using the *Hph*MX4 cassette amplified from plasmid pAG32 [34] by using primers DELREV3FW (gttcataaaacgctggaagtaaaaattagggcatcctttaaaaggCCTTGACAGTCTTGACGTGC) and DELREV3RV (aagtaatcgaatacatttttcaataatcttctgctgggctgggatCGCACTTAACTTCGCATCTG), each of which contains a 5′ stretch complementary to a sequence internal to *REV3* ORF and a 3′ stretch, in uppercase, complementary to the extremities of the *Hph*MX4 cassette. Disruptants were as resistant to 250 µg/ml hygromycin B. Correct disruption was confirmed by PCR.

*REV7* and *REV1* were disrupted in strain DWM-5A9 by using the *Kan*MX4 cassette. The disrupted *rev7::Kan^R^* and *rev1::Kan^R^* loci were amplified from strain BY4742 *rev7Δ* and BY4742 *rev1Δ*, respectively, by using primers external to the loci: REV7-Fw and REV7-Rv, or REV1-Fw and REV1-Rv. Disruptants were selected on YP supplemented with 200 µg/ml geneticin and disruption was confirmed by PCR.

### Transformation of yeast strains

Yeast strains were transformed by lithium acetate methods [47]. We used the “Quick and easy method” (http://home.cc.umanitoba.ca/~gietz/Quick.html) in case of transformation with centromeric or episomal (multicopy) plasmids, and the “Best Method” (http://home.cc.umanitoba.ca/~gietz/method.html) in case of disruption cassettes.

### Determination of mitochondrial and nuclear mutability

*Petite* frequency was determined as previously reported [48]. To determine the effect of antioxidant agents on the extended mtDNA mutability, wild-type and mutant strains, were grown on liquid SCD medium supplemented with 40 µM dihydrolipoic acid (Sigma) or 10 µM MitoQ (a kind gift from Luigi Palmieri, University of Bari, Italy) for two 24-hour growth cycles. Dihydrolipoic acid was supplemented from a 40 mM stock solution in ethanol, while MitoQ was supplemented from a 5 mM stock solution in DMSO. Control experiment in which equal amounts of ethanol (0.1%) or DMSO (0.2%) were added to untreated cells was done in parallel. For each strain/condition at least three independent experiments were performed on three independent clones. Statistical analysis of *petite* frequency was performed by a two-tailed t-test.

Ery^R^ mutant frequency was determined as previously reported [48]. Briefly, 15 independent colonies, isolated on SC medium supplemented with 2% glucose, were inoculated into 2 ml of SC liquid medium supplemented with 2% ethanol and grown to saturation (approximately 48 h). Cultures were then plated on YPAEG-Ery medium and dilutions were plated nonselectively on YP medium supplemented with 3% glycerol (300–500 colonies) for a total cell count. After 7 days incubation, Ery^R^ mutant frequency was determined by the method of the median [49]. Each experiment was repeated at least three times on two independent clones for each strain. Statistical analysis of Ery^R^ mutant frequency was performed by a one-tailed t-test.

Nuclear point mutability was evaluated by measuring the mutation rate of Can^R^ mutants in strain W303-1A CAN^S^, bearing the wt copy of *CAN1*, as previously reported [50], except that colonies were grown on SC medium supplemented with 2% glucose. Mutation rates were calculated using median values according to Lea and Coulson [49] by using the program FALCOR [51]. Rates are given as mean of four independent experiments in each of which 10 independent colonies for each clone were tested. Unpaired two-tailed t-test was used to evaluate the statistical significance.

In every test, frequencies or rates were considered significantly different when p<0.05.

### Northern blot and semiquantitative RT-PCR

Total RNA was extracted from 10 ml of culture at a concentration of 1 OD_600_/ml, according to [52]. For Northern blot analysis, 25 µg of RNA were blotted according to [53]. The probes for hybridization were obtained by purifying the 1 Kb *Eco*RV fragment of *REV3* and by amplifying *REV7* with primers REV7N-Fw (gggtagagaagtggctgagg) and REV7N-Rv (ctcgccctcttcatattgag). Probes were labeled using [α-32P]dCTP and the kit ‘Rediprime II DNA Labelling System’ (GE Healthcare). Signals were acquired after different time exposures with Kodak films or with PhosphorImager and quantified by ImageQuant software (Molecular Dynamics).

For RT-PCR, 1.5 µg of RNA were treated with DNase (Invitrogen), precipitated and converted to cDNA with ProtoScript® M-MuLV First Strand cDNA Synthesis Kit (NEB) by using oligod(T). Semiquantitative PCR was performed by amplifying a fragment of *REV1* cDNA with primers REV1rtFW (gtatattccagccgatcaaattcc) and REV1rtRV (cgtttggaagcctgaatgatcc) and, as control, a fragment of *TAF10* DNA with primers FWTAF10 (atattccaggatcaggtcttccgtagc) and RVTAF10 (gtagtcttctcattctgttgatgttgttgttg). PCR samples were taken after 20, 23, 26, 29, 32 and 35 cycles, and aliquots of equal volume was loaded on a 0.8% agarose gel. Images were acquired by Kodak Digital Science D120 zoom digital camera, converted to gray scale with Photoshop; signals were quantified through Multianalyst software (Molecular Dynamics). To avoid saturation, signals were considered only if the signal deriving from the PCR amplification with 3-cycle less was 6–8-fold lower. Both in the case of Northern analysis and in the case of RT-PCR, relative levels of mRNA were quantified by comparison with a house-keeping gene signal (*ACT1* or *TAF10*). All experiments were performed three times.

### Fluorescence microscopy

Cells were grown in solid SC medium without methionine and supplemented with 2% glucose. After 24 hours, cells were resuspended in Tris–HCl 50 mM pH 7.5, incubated with 1 µg/ml DAPI (4′,6-diamidino-2-phenylindole) for 15 min on ice and observed with a 100× magnification using DAPI or FITC filters. Pictures were taken with constant parameters (100 ms exposure for DAPI and 250–500 ms exposure for GFP with Nikon ACT-1 acquisition software) and processed to optimize contrast and brightness with Adobe Photoshop 7.0.

## Results

### Pol zeta overexpression rescues the mtDNA mutability induced by specific *mip1* mutations

**Figure 1:**
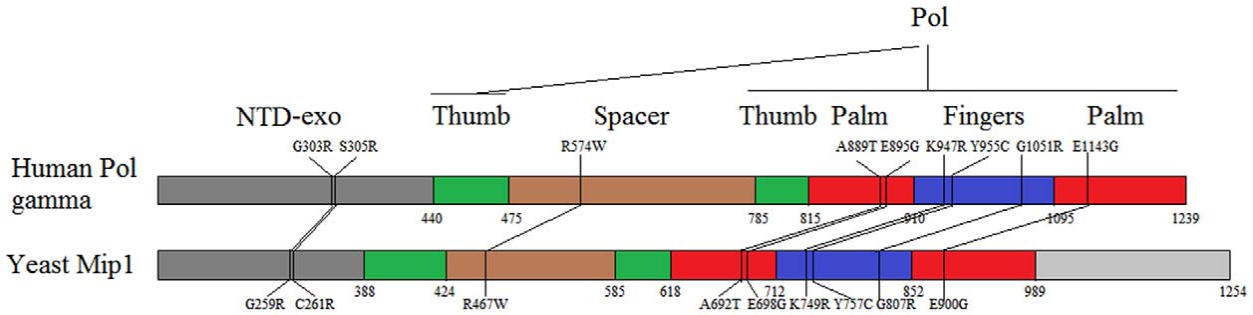
Linear representation of human Polg and yeast Mip1 with the mutations used in this study. NTD-Exo = N-terminal domain- 3′-5′ exonuclease domain. Pol = polymerase domain.

We have previously constructed a set of Saccharomyces cerevisiae *mip1* mutant alleles, corresponding to *POLG* pathological mutations, and determined their effects on mtDNA extended and point mutability (Table 1) [19]–[21], [23]. These mutant alleles localized in different Mip1 domains, displayed different *in vivo* and *in vitro* defects and behaved either as dominant or recessive (Table 1 and Figure 1).We analyzed the effect of overexpression and deletion of Pol zeta and Rev1 on the mtDNA mutability induced by these *mip1* mutations, based on the recent finding that these polymerases were found in mitochondria, where Pol zeta and Mip1 belong to the same epistatic pathway [9].

**Table 1:** *POLG* human pathological mutations, associated syndromes, *mip1* equivalent mutations analyzed in this study and phenotypes of *mip1* mutant strains.

Human mutation	Pathology	Yeast mutation	Domain	*In vivo* phenotype: *petite* frequency	*In vivo* phenotype: Ery^R^ frequency (fold increase relative to wt)	Dominance/recessivity	Polymerase activity (compared to wt)	Soluble protein levels (compared to wt)	Rescue by treatment with antioxidants
G303R	Alpers	G259R	Exo	≈80%	NT	Recessive	NT	NT	NT
S305R	Alpers	C261R	Exo	≈85%	≈6	Recessive	Reduced	NT	Slight
R574W	PEO+myopathy/dysphagia Alpers	R467W	Linker	≈60%	≈4	Recessive	As wt(Reduced processivity)	As wt	No
A889T	PEO	A692T	Pol	≈80%	≈4	Slightly dominant^1^	≈30%	As wt	No
E895G	Myopathic MDS	E698G	Pol	100%	ND	Dominant	NT	NT	Strong
K947R	PEO, ovarian failure	K749R	Pol	100%	ND	Dominant	NT	NT	Strong
Y955C	PEO+/−Parkinsonism, Premature ovarian failure, Alzheimer's disease	Y757C	Pol	100%	ND	Dominant	<0.03%	NT	Strong
G1051R	arPEO, SANDO	G807R	Pol	≈25%	≈12	Recessive	70–80%	10–15%	No
E1143G	Neutral polymorphism but may modulate disease mutations	E900G	Pol	≈6%	≈2	Recessive	100–130%	30–40%	NT

NT: not tested.

ND: not detectable.

1 The mutation is slightly dominant in yeast, where the *petite* frequency in heterozygosis is approximately 1.5-fold compared to the wild type. In humans, the equivalent mutation has been associated either to sporadic PEO when it is *in trans* with mutation R579W, suggesting a recessive trait [80] and to PEO with tremor and peripheral neuropathy when it is *in cis* with E1143G SNP and *in trans* with wt *POLG*, suggesting a dominant trait [81].

*REV3* and *REV7* were cloned in the multicopy plasmid YEplac195 and introduced i) in the strain DWM-5A (*mip1Δ*) transformed with a number of recessive *mip1* mutant alleles (haploid condition) and ii) in the strain W303-1B (*MIP1*) transformed with dominant *mip1* mutant alleles (heteroallelic condition). Extended mtDNA mutability was first determined in these transformants by analyzing the frequency of spontaneous *petite* mutants.

**Figure 2:**
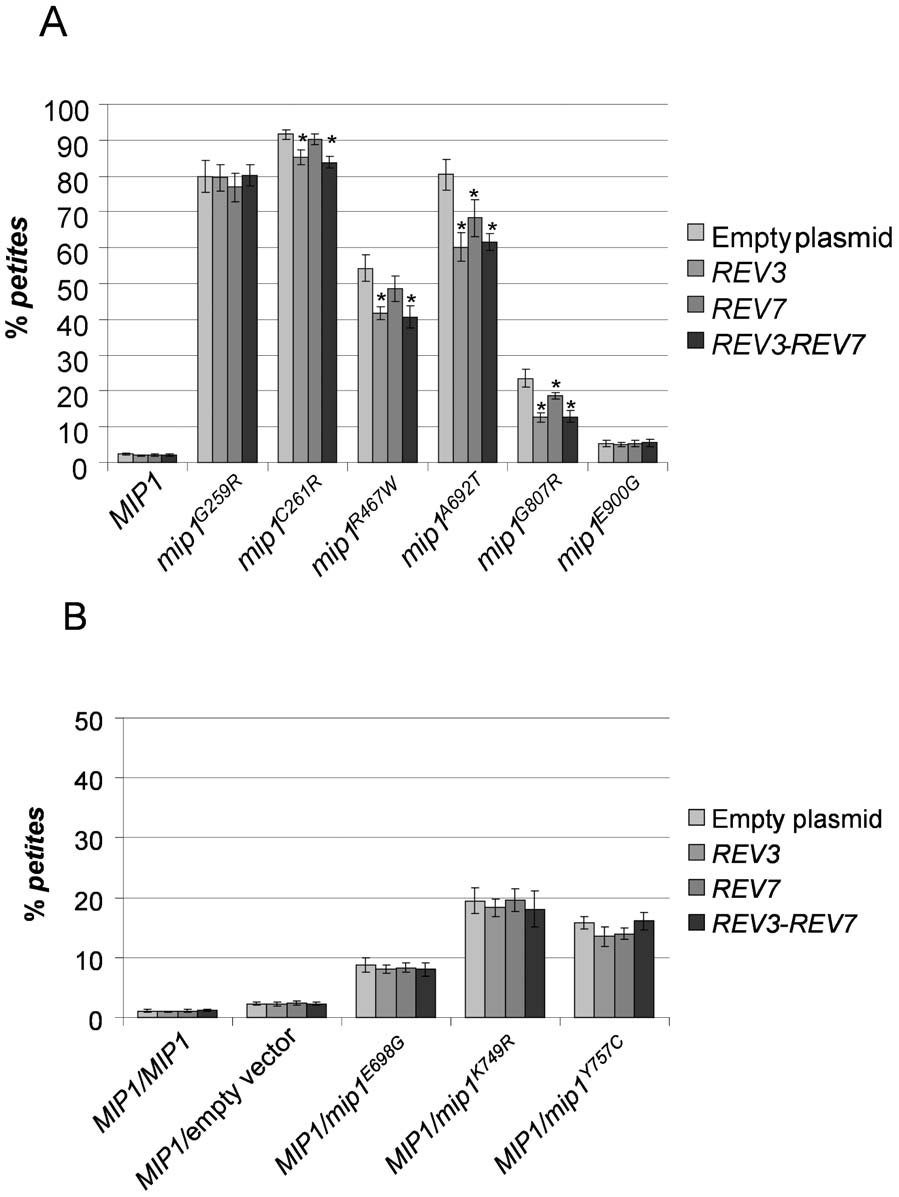
*Petite* frequency of *mip1* strains overexpressing *REV3*, *REV7*, or both. *Petite* frequency was measured as reported in Materials and Methods on three independent clones for each strain. All values are means at least of three independent experiments. (A) Haploid mutant strains harboring *mip1* recessive mutant alleles. (B) Heteroallelic strains harboring *MIP1* wt allele and *mip1* dominant mutant alleles. For each *mip1* allele, ^*^ indicates that the *petite* frequency is significantly lower (p<0.05) than that of strain transformed with the empty plasmid (YEplac195).

In presence of overexpressed *REV3*, the level of *petite* mutants decreased; the rescue was mutation specific, being null for some mutations and significant for other ones (Figure 2). The strongest effect was observed for haploid strains bearing mutations G807R (50% reduction) and A692T (25% reduction). The *REV7* overexpression, on the contrary, had slighter effects, which however were significant for mutations A692T and G807R (Figure 2A). In heteroallelic strains harboring wt *MIP1* and dominant *mip1^E698G^*, *mip1^K749R^* or *mip1^Y757C^* mutant alleles (Figure 2B) no rescue was observed in the presence of either *REV3* or *REV7*.

**Figure 3:**
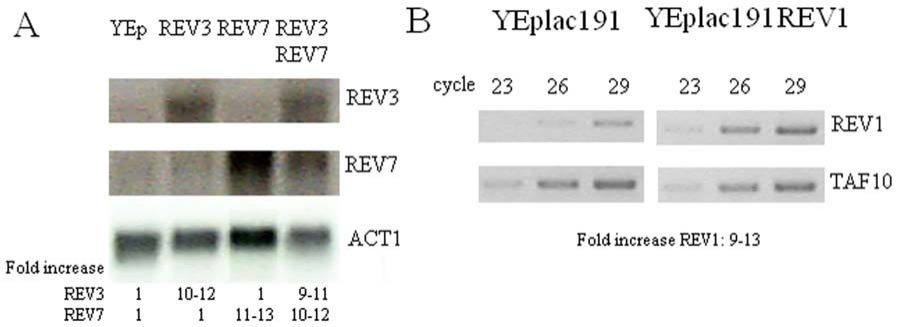
mRNA levels in overexpressing strains. A. Northern blot mRNA expression of *REV3* and *REV7* in strains transformed with YEplac195 empty plasmid (1^st^ lane), YEplac195-*REV3* (2^nd^ lane), YEplac195-*REV7* (3^rd^ lane) and YEplac195-*REV3/REV7* (4^th^ lane). Numbers indicate the fold increase of *REV3* and *REV7* levels in overexpressing strains. mRNA of the *ACT1* gene was used as a control. B. RT-PCR on *REV1* in strains transformed with YEplac81-*REV1* and YEplac181 empty plasmid. cDNA of the *TAF10* gene was used as control. Numbers over the gel indicate the number of PCR cycles, number under the gel indicate the fold increase of *REV1* levels in overexpressing strains.

We also evaluated the combined effects of *REV3* and *REV7* overexpression by cloning the two genes together in the YEplac195 plasmid. Northern blot analysis indicated that *REV3* and *REV7* were expressed at high level (10–12 fold increase of mRNA levels) when cloned either alone or together, demonstrating that their concomitant presence in the plasmid did not affect the level of their expression (Figure 3A). Overexpression of whole Pol zeta had the same effect than overexpression of Rev3 (Figure 2), suggesting that Rev3 alone was responsible of the observed rescue or that the physiological levels of Rev7, which is more abundant than Rev3 [54], were sufficient to ensure the highest level of rescue. However the slight decrease in *petite* frequency obtained overexpressing Rev7 alone suggests, as demonstrated below, that also Rev7 participates to the rescue and that the *petite* mutants' reduction observed is most likely due to a slight increase of the whole Pol zeta.

### Effect of *REV3* deletion on extended and point mtDNA mutability

We next investigated the effect of *REV3* deletion on mtDNA stability. No difference was observed between *REV3* and *rev3Δ* regarding the frequency of *petite* mutants in strains harboring either wt *MIP1* allele or mutant *mip1* alleles, indicating that the *REV3* deletion produced no effect on extended mutability (data not shown).

However, deletion of *REV3* resulted in alteration of mtDNA point mutation, with 2.5 fold increase of Ery^R^ mutant frequency in the *MIP1* wt strain, in agreement with results reported by Kalifa and Sia [10] (Table 2). In addition, strains carrying *mip1^C261R^*, *mip1^A692T^* and *mip1^G807R^* mutant alleles showed a further increase of Ery^R^ mutation (an increase of 3.9-, 3.3- and 5.1-fold, respectively) (Table 2), indicating that mutations in *MIP1* and deletion of *REV3* have a synergistic effect in rising point mutability. Furthermore, the ratios of Ery^R^ mutant frequencies of *rev3Δ* strains to *REV3* wt strains parallelized the mutator phenotype of the *mip1* mutant allele, being the lowest in *mip1^A692^* ^T^ strain showing a moderate increase of Ery^R^ frequency compared to wt *MIP1* strain (approximately 3.4-fold), and being the highest in the strain *mip1^G807R^* showing an increase of 8.8 times the Ery^R^ frequency of *MIP1* strain (Table 2). Therefore, Pol zeta is not physiologically involved in the maintenance of full mtDNA but is required for the fidelity of the mtDNA replication.

**Table 2:** Ery^R^ mutant frequency in wt and *rev3Δ* strains transformed with wt or mutant *mip1* alleles.

	Ery^R^ per 10^7^ cells:wt strain	Fold increasemutant *mip1*/wt *MIP1*	Ery^R^ per 10^7^ cells:*rev3Δ* strain	Fold increase *rev3Δ* strain/wt strain
***MIP1***	1.5±0.3	1.0	3.7±0.6	2.5
***mip1^C261R^***	5.6±0.8	3.7^*^	22.0±2.9	3.9^*^
***mip1^A692T^***	5.1±0.6	3.4^*^	16.6±2.7	3.3
***mip1^G807R^***	13.2±1.9	8.8^*^	67.9±9.4	5.1^*^

Ery^R^ mutant frequency in wt and *rev3Δ* strains transformed with wt or mutant *mip1* alleles was determined as reported in Materials and Methods. “Fold increase mutant *mip1*/wt *MIP1*” was obtained by dividing the Ery^R^ frequency related to each *mip1* allele by the Ery^R^ frequency related to *MIP1* wt allele. “Fold increase *rev3Δ* strain/wt strain” was obtained by dividing, for each *mip1* allele, the Ery^R^ frequency of *rev3Δ* strain by that of wt strain. * indicates that the fold increase is significantly higher (p<0.05) than that of the *MIP1* wt allele.

### Effects of *REV1* overexpression

**Figure 4:**
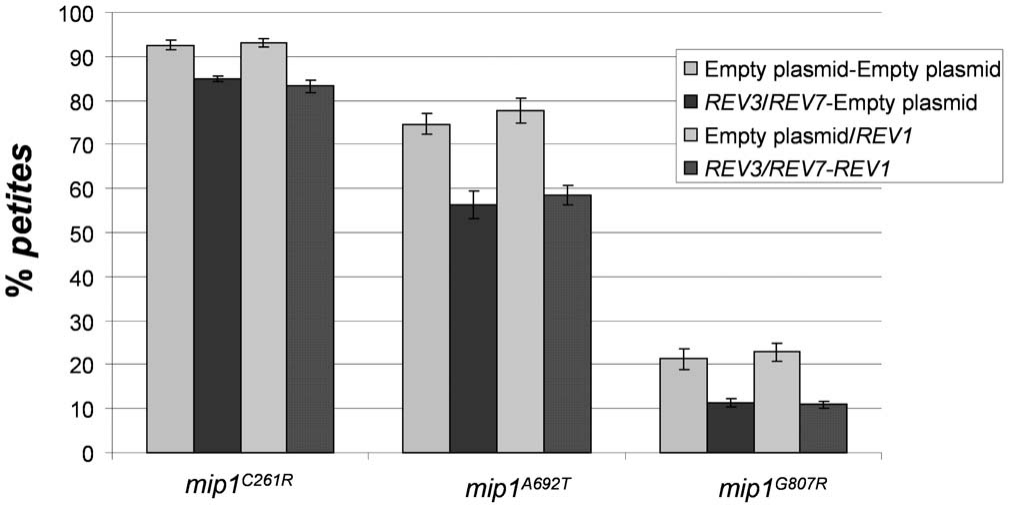
*Petite* frequency of *mip1* mutant strains overexpressing Pol zeta, Rev1, or both. *Petite* frequency was measured as in Figure 2.

Rev3 subunit of Pol zeta binds to Rev1, and the complex Pol zeta-Rev1 increases the efficiency of translesion synthesis by Pol zeta [8]. We tested whether overexpression of *REV1*, together with overexpression of Pol zeta, could further reduce the *petite* frequency due to *mip1^C261R^*, *mip1^A692T^* and *mip1^G807R^* mutant alleles. *REV1* was cloned in the multicopy plasmid YEplac181 and introduced in strains overexpressing both *REV3* and *REV7* and carrying wt *MIP1*, *mip1^C261R^*, *mip1^A692T^* and *mip1^G807R^* mutant alleles. RT-PCR analysis confirmed that *REV1* was 9–13-fold overexpressed in transformant cells (Figure 3B) and that *REV3* and *REV7* were overexpressed at the same levels (10–12 fold overexpression) as in strain not harboring YEplac181-*REV1* (data not shown). Together with *REV1*, we overexpressed both *REV3* and *REV7*, and not *REV3* alone, because it is known that Rev1 can recruit Rev7 through a Rev3 independent Rev1-Rev7 binding thus depleting the levels of Rev7 [54]. *REV1* overexpression did not alter the frequency of *petite*, both in presence and absence of overexpressed Pol zeta (Figure 4). This result indicates that either the physiological levels of *REV1* are sufficient for the rescue by Pol zeta overexpressed or that Rev1 is not involved. Moreover, we observed that overexpression of both Pol zeta and Rev1, but not of either Pol zeta or Rev1 individually, resulted in a significant reduction of the Ery^R^ mutant frequency, in particular for the strain carrying the mutation G807R (Table 3). The results described here are symmetrical to those obtained previously by Kalifa and Sia [10], who showed an increase in Ery^R^ mutant frequency when *REV1*, *REV3* or *REV7* were disrupted

**Table 3:** Ery^R^ mutant frequency in *mip1* mutant strains overexpressing *REV3*, *REV7* and/or *REV1*.

	Empty plasmid- empty plasmid	*REV3*/*REV7*- empty plasmid	Empty plasmid-*REV1*	*REV3*/*REV7*-*REV1*
***MIP1***	1.4±0.2	1.3±0.3	1.6±0.3	1.0±0.3
***mip1^C261R^***	4.9±0.7	4.4±0.7	4.2±0.4	1.9±0.2*
***mip1^A692T^***	4.3±0.5	4.5±0.6	4.7±0.6	2.3±0.3*
***mip1^G807R^***	16.7±2.1	15.2±1.8	16.4±3.7	6.5±1.1*

Ery^R^ mutant frequency (per 10^7^ cells) was determined as reported in Materials and Methods. For each *mip1* strain,

* indicates that the Ery^R^ mutant frequency is significantly lower (p<0.05) than that of strain harboring both empty plasmids.

### Rescue of extended mutability by Rev3 overexpression is Rev7 dependent and Rev1 independent

We tested whether the rescue by Rev3 overexpression was dependent on the physiological levels of Rev7 and Rev1. The deletion of *REV1* or *REV7* had no effect on *petite* mutability (data not shown). However, the rescue of *petite* mutability in *MIP1* mutants, exerted by overexpressed Rev3, occurred only when Rev7 was present (Table 4), indicating that the whole Pol zeta participated in the rescue. We furthermore observed that overexpression of *REV7* in a *rev3Δ* strain did not reduce *petite* frequency both in wt *MIP1* and in *mip1* mutant strains (data not shown), corroborating this hypothesis. Unlike Rev7, Rev1 was dispensable (Table 4), thus supporting the hypothesis reported in the previous paragraph that Rev1 did not participate in the rescue of the extended mutability.

**Table 4:** *Petite* frequency in *mip1* mutant strains deleted of *REV1* or *REV7* and overexpressing *REV3* and *REV7*.

	*mip1^C261R^*			*mip1^A692T^*			*mip1^G807R^*		
	Empty plasmid	*REV3*	*REV3*/*REV7*	Empty plasmid	*REV3*	*REV3*/*REV7*	Empty plasmid	*REV3*	*REV3*/*REV7*
***rev1Δ***	89.4	81.3*	81.6*	78.6	60.0*	62.9*	23.8	12.2*	13.4*
***rev7Δ***	91.5	89.4	82.7*	79.2	80.2	57.3*	24.0	24.6	13.1*

*Petite* frequency was measured as reported in Figure 2. For each *mip1* mutant *rev1Δ* or *rev7Δ* strain,

* indicates that the frequency is significantly different (p<0.05) compared to that of strain transformed with the YEplac195 empty plasmid.

### Combined action of *REV3* overexpression and other chemical and genetic rescues

**Figure 5:**
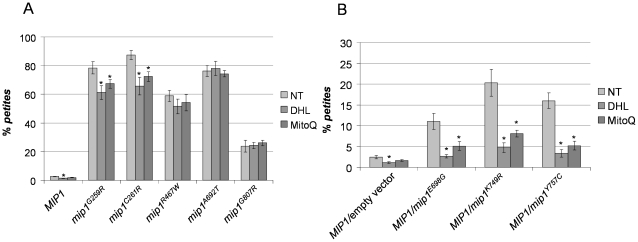
*Petite* frequency of *mip1* strains treated with dihydrolipoic acid 40 µM (DHL) or 10 µM MitoQ. *Petite* frequency was measured as reported in Materials and Methods on three independent clones for each strain at least twice. (A) Haploid mutant strains harboring *mip1* recessive mutant alleles. (B) Heteroallelic strains harboring *MIP1* wt allele and *mip1* dominant mutant alleles. For each *mip1* strain, * indicates that the *petite* frequency is significantly lower (p<0.05) than that of non-treated (NT) strain.

We have shown previously that a decrease of reactive oxygen species and/or an increase of the mitochondrial dNTP pool reduced the mtDNA extended mutagenesis induced by pathological Pol gamma mutations in yeast [19]. Several mutations in *MIP1*, but not all, were rescued, albeit at different extent, by treatment with dihydrolipoic acid or with the mitochondrial specific ROS scavenger MitoQ [21]. To know whether this effect could be increased by *REV3* overexpression the *petite* frequency was measured either in haploid (Figure 5A) or in heteroallelic strains (Figure 5B) treated with these molecules. We found that there was a negative correlation between the rescue exerted by ROS scavengers and the rescue caused by *REV3* overexpression (Figure 2 and Figure 5).

In fact, in haploid strains harboring A692T and G807R mutations, whose effect was reduced by overexpression of Rev3, no rescue was exerted by dihydrolipoic acid or MitoQ. In contrast, ROS scavengers strongly mitigated the effect of *mip1* mutations E698G, K749R, Y757C, that are insensitive to Rev3 overexpression.

The only exception was mutation C261R, whose effect was slightly rescued both by treatment with ROS scavengers and by Rev3 overexpression (Fig. 2A and Fig. 5A). In this case, the additive effect of Pol zeta overexpression and ROS scavengers (data not shown) suggested that two different mechanisms were involved in the reduction of *petite* mutability observed.

The second mechanism able to rescue the *mip1* induced mtDNA extended mutability is the increase of the dNTP pools. In *S. cerevisiae* an increase of the dNTP pool was obtained either by overexpressing *RNR1* gene, which encodes the large subunit of the ribonucleotide reductase or by deleting *SML1* gene, which encodes an inhibitor of the latter activity [55]. It has been shown that overexpression of *RNR1* or deletion of *SML1*, reduced the *petite* frequency in strains harboring specific *MIP1* mutations [20], [26], [39], [55].

**Figure 6:**
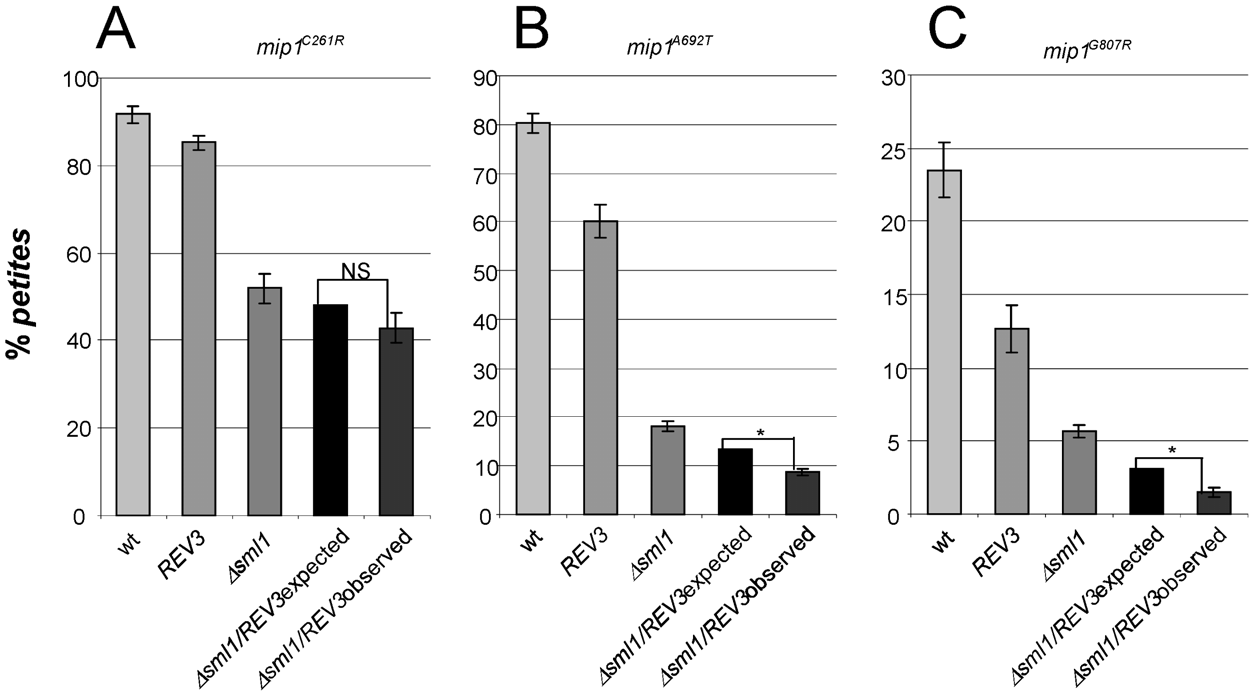
*Petite* frequency of *mip1^C261R^*, *mip1^A692T^* and *mip1^G807R^* strains disrupted in *SML1* and overexpressing *REV3*. In each graph the following *petite* frequencies are reported: *mip1* strains transformed with YEplac195 empty plasmid (wt) or with YEPlac195-*REV3* (*REV3*); *mip1 sml1Δ* strains transformed with YEplac195 empty plasmid (*sml1Δ*) or with YEplac195-*REV3* (*sml1Δ*/*REV3* observed). In addition, the penultimate bar represents the *petite* frequency of *sml1Δ* strain transformed with YEplac195-*REV3* that would be expected if the effects of the overexpression of *REV3* and of the deletion of *SML1* were additive (*sml1Δ*/*REV3* expected). * indicates that the observed frequency is significant lower than the expected one. NS: not significant.

To evaluate the relationship between the rescue exerted by *REV3* overexpression and the rescue exerted by increasing the dNTP pools, we deleted *SML1* in strains harboring *mip1^C261R^*, *mip1^A692T^* and *mip1^G807R^* mutant alleles, in presence of overexpressed *REV3*. We observed that, for every mutation studied, the two processes act synergistically (Figure 6). For example, in strain carrying *mip1^A692T^* mutant allele the overexpression of *REV3* reduced the *petite* frequency of approximately 1.3-fold, while deletion of *SML1* reduced the *petite* frequency of 4.4 fold: together, the reduction was 9.1-fold. The synergistic effects were even stronger in the case of strain harboring *mip1^G807R^* mutation (1.9-fold when *REV3* was overexpressed, 4.1-fold when *SML1* was deleted, 15.7-fold together). It is likely that the observed synergy is due to an increase of dNTP pools, which are the substrate for both Mip1 and Pol zeta.

### Construction of a mutant Rev3 which rescues mtDNA mutability without affecting nuclear mutability

**Figure 7:**
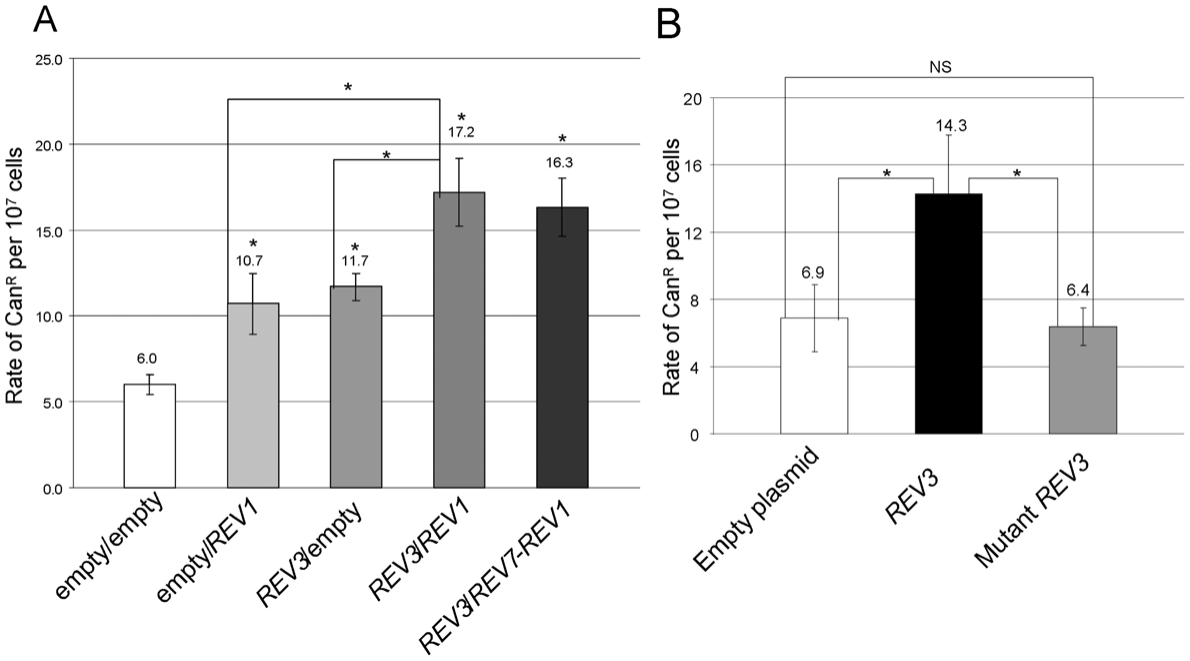
Rate of Can^R^ mutants. Rate of Can^R^ mutants (A) in wt strains overexpressing *REV3*, *REV7* and/or *REV1* or (B) wt *REV3* and mutant *REV3*, encoding for the Rev3 protein harboring the 504–508 AAAAA stretch. Rate of Can^R^ mutants was measured as indicated in Materials and Methods on three independent clones. If no stated differently, * indicates that the rate is significantly different from that of strain transformed with the empty plasmid(s). NS: not significant.

It was previously shown that in UV-treated cells, enhanced expression of *REV3* led to increase of nuclear point mutability, measured as increase in the rate of *arg4–17* reversion. Pol zeta mutagenic function is dependent on Rev1 protein [38]. We measured nuclear mutability in cells transformed with *REV3* and/or *REV1* overexpressing plasmids to determine whether increased levels of Pol zeta/Rev1 exerted mutagenic effect as a consequence of a general mechanism associated to the translesion bypass. The overexpression of Rev3 or Rev1 caused an approximately two-fold increase of nuclear point mutability, measured as rate of Can^R^ mutants. When both Pol zeta and Rev1 were overexpressed, the rate of Can^R^ mutations increased approximately 3-fold (Figure 7). Thus, overexpression of Pol zeta is useful to reduce the mtDNA instability caused by specific mutations in *MIP1* but is detrimental to spontaneous nuclear mutability.

**Figure 8:**
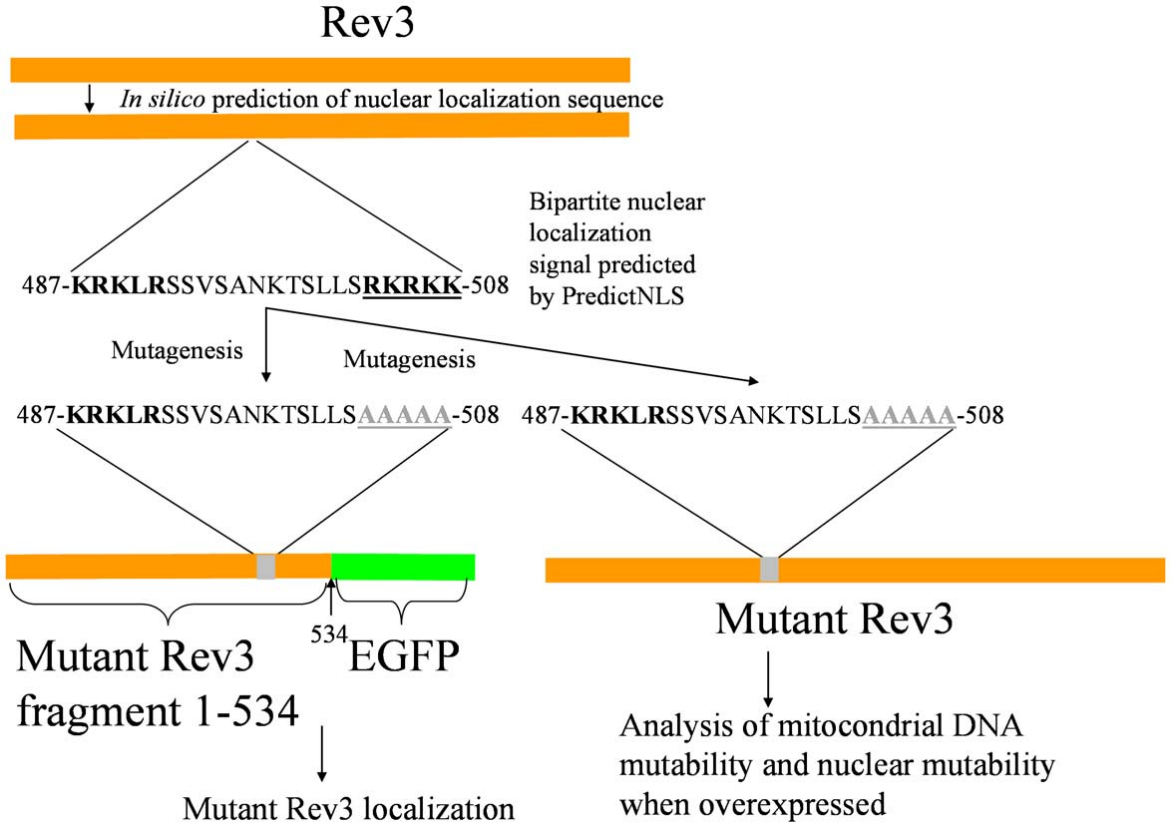
Position and sequence of the bipartite nuclear localization sequence. The stretch RKRKK was mutagenized to AAAAA to obtain a mutant Rev3 fragment fused with EGFP for protein localization and a mutant Rev3 isoform for analysis of mtDNA mutability and nuclear point mutability.

A Rev3 mutant which could enter into mitochondria, but not in the nucleus is expected to reduce the mitochondrial mutability without increasing nuclear mutability. To this end, we thought of designing a *REV3* gene whose product could not enter the nucleus. We searched inside Rev3 for the potential nuclear localization signal, by using the PredictNLS server (http://cubic.bioc.columbia.edu/services/predictNLS/). Rev3 protein is predicted to possess a unique bipartite nuclear localization signal encompassing amino acids 487–508 (Figure 8). Both upstream and downstream basic amino acid stretches composing the bipartite signal are critical for nuclear import [56].

To evaluate the localization of Rev3, we first cloned the whole coding sequence either in a centromeric plasmid or in a multicopy plasmid in frame with the EGFP gene. In both cases no GFP signal was detected in transformant cells, as it was found previously in high throughput analysis [57]. To overcome this problem, a shorter Rev3 fragment encompassing residues 1–534, which include the bipartite signal, was cloned in frame with EGFP. In this case, in transformant cells we observed a fluorescent signal corresponding to Rev3, localized in the nucleus. This was confirmed by DAPI staining of mitochondrial and nuclear DNA (Figure 6). Both in *rho^+^* strain, where DAPI stained both mtDNA and nuclear DNA, and in the *rho^0^*, where DAPI stained only nuclear DNA, the GFP signal co-localized with the nuclear DAPI signal.

**Figure 9:**
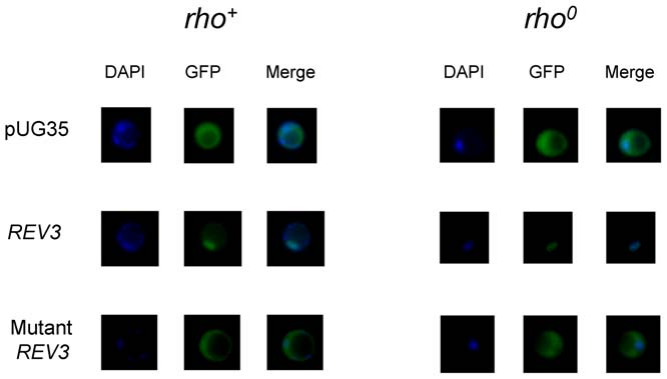
Localization of Rev3. Localization of Rev3 and mutagenized Rev3 fused with EGFP in (A) a *rho^+^* strain and in (B) a *rho^0^* strain. Images have been acquired and adjusted as reported in Materials and Methods. In *rho^+^* strain DAPI stains both the nuclear DNA (bigger spot) and the mtDNA (small spots in the cell periphery). In *rho^0^* strain, which is depleted of mtDNA, DAPI stains the nuclear DNA only.

To obtain a version of Rev3 unable to migrate in the nucleus, we changed the 504–508 RKRKK downstream stretch to AAAAA (Figure 8). When Rev3 fragment encompassing amino acid 1–534 and harboring the 504–508 AAAAA stretch was fused with EGFP, the fluorescent signal corresponding to the fusion protein localized in the cytoplasm only (Figure 9). The AAAAA mutation was then introduced in the full *REV3* gene, cloned in the multicopy plasmid YEplac195 and its effect was evaluated in strains harboring *mip1^A692T^* or *mip1^G807R^*. In the presence of overexpressed mutant Rev3, the mtDNA extended mutability decreased at the same extent as achieved in the presence of overexpressed wild type Rev3, indicating that the alteration of the nuclear signal did not affect the ability of Rev3 of operating in mitochondria (Table 5). If the mutated version of the enzyme did not enter the nucleus, then it was expected that its overexpression did not cause an increase in nuclear mutability. Indeed, in the presence of overexpressed mutant Rev3 the nuclear point mutability, measured as rate of Can^R^ mutants accumulation, did not increase (Figure 7).

**Table 5:** *Petite* frequency in *mip1* strains overexpressing *REV3* or the mutagenized form of *REV3*.

	Empty plasmid	*REV3*	Mutant *REV3*
*MIP1*	2.4±0.3	2.0±0.2	2.2±0.2
*mip1^A692T^*	80.4±4.3	60.2±4.1*	63.2±3.6*
*mip1^G807R^*	23.5±2.4	12.6±1.4*	11.6±1.7*

*Petite* frequency was measured as reported in Figure 2. For each *mip1* strain,

* indicates that the frequency is significantly different (p<0.05) compared to that of strain transformed with the YEplac195 empty plasmid. The differences between strains overexpressing wt *REV3* or mutant *REV3* are not significant.

## Discussion

In this study we showed that increased levels of Rev3, encoding the catalytic subunit of Pol zeta, reduces both the mtDNA extended mutability and the point mutability caused by specific mutations in Mip1, the *S. cerevisiae* mtDNA polymerase. The rescue of mtDNA extended mutability requires overexpression of the catalytic subunit Rev3, but not that of the accessory subunit encoded by *REV7*. However, a basal level of Rev7, which is known to be expressed more than Rev3 [54], is necessary, because in a *rev7Δ* strain the rescue does not occur.

Rev1, which interacts with Pol zeta for efficient bypass and extension past the DNA lesion, is not required to rescue mtDNA extended mutability, suggesting that Pol zeta can function independently of Rev1 in mitochondrion. In contrast, Rev1 overexpression is necessary for the rescue of point mutability mediated by Rev3. These observations suggest that the rescues mediated by Rev3 overexpression are driven by two different mechanisms: i. the rescue of mtDNA extended mutability involving only Pol zeta and ii. the rescue of mtDNA point mutability for which both Pol zeta and Rev1 are required. These results are consistent with previous findings which indicate that the instability of mitochondrial DNA in general is caused by mechanisms different from those producing single point mutations [19].

Deletion of *REV3* did not influence the mtDNA extended mutability (*petite* frequency), both in the wt strain and in *mip1* mutant strains. In contrast, *REV3* deletion caused an increase of point mutability. Therefore Pol zeta, in mitochondria, functions in a pathway less mutagenic than Mip1, supporting the hypothesis of Kalifa and Sia [10]. Moreover, strains carrying *mip1* mutant alleles were themselves mutators, i.e. showed an Ery^R^ mutant frequency higher than that of the wt strain. The observation that the higher the mtDNA point mutability of the mutant strain, the higher the effect of *REV3* deletion, indicates that mutator Mip1 isoforms are more sensitive to the absence of Rev3 than the wild type one and is coherent with that hypothesis.

Until now, it has not been yet elucidated the physiological role of Pol zeta in mitochondria. Rev3, that is expressed at basal levels during the whole vegetative cell cycle and only moderately (∼2-fold) overexpressed when cells are treated with UV, is expressed at levels comparable or higher (∼18-fold) than those obtained in this study during the late phase of sporulation [58]. Interestingly, maintenance of a full non-mutated mtDNA genome is fundamental in meiosis, since respiratory-deficient diploid strains are unable to sporulate or show increased levels of dead spores [59], [60]. A possible interpretation is that increased levels of Pol zeta could have importance in the maintenance of wt mtDNA during sporulation.

Regarding extended mutability, some *MIP1* mutations equivalent to human pathological mutations were rescued by overexpression of Rev3 while others were not. Y757C, E698G and K749R, dominant mutations located in the polymerase domain, together with the recessive mutation in the exonuclease domain G259R, belong to this second group. These mutations are rescued by treatment with antioxidant molecules such as dihydrolipoic acid and MitoQ. Biochemical properties are available only for human Y955C mutation, equivalent to yeast Y757C, that strongly reduces the polymerase activity (<0.03% compared to the wild type), the processivity and the nucleotide binding affinity, but does not affect the DNA binding affinity [61], [62]. It was previously reported also that human Pol gamma harboring the Y955C mutation increases the *in vitro* incorporation of 8-oxo-dGTP, the most abundant oxidized nucleotide in mitochondria, and the translesion synthesis opposite to an 8-oxo-dG residue [63]. The last observation suggests that, although most of the mtDNA extended mutability by Y757C mutation is caused by stalled replication due to strongly reduced polymerase activity [61], [62], [64], [65], the mtDNA damage can be partially induced also by the accumulation of oxidized bases. In addition, for heteroallelic strains containing dominant mutations, it is possible that replication stalling by mutant Mip1 itself causes oxidative damage on mtDNA, which can partially block replication by wt Mip1. Independently of the mechanisms, it is known that there is a correlation between mtDNA extended mutability and oxidative stress: *petite* frequency is higher in strains with a reduced antioxidant potential [66], in strains treated with hydrogen peroxide [67] and in strains deleted both in *MSH1* and in *OGG1*, encoding the mitochondrial glycosylase/lyase that specifically excises 8-oxo-dG residues in mtDNA [68].

Differently from Y757C, E698G, K749R and G259R, mutations C261R, R467W, A692T and G807R are rescued by Rev3 overexpression. These mutations are known to reduce the polymerase activity and/or the processivity of Mip1 [20], [27]. Yeast C261, equivalent to human S305, is part of a loop-helix orienter module (amino acids 304–316) that creates steric constraints leading to a destabilization of the template strand and a modification in the arrangement of the primer strand [27]: this subdomain in the 3′–5′ exonuclease plays a crucial role in the DNA polymerase function with no major effect on the exonucleolytic activity. yG807, equivalent to hG1051, is part of a partitioning loop (amino acids 1050–1095) [12] that modulates the partitioning of the primer strand between the polymerase and the exonuclease active sites by forming stable contacts with correctly base-paired primer-template DNA and destabilizing primer-template DNA that contains mispairs [69] This subdomain is adjacent to the orienter module, whose role is to position correctly the partitioning loop. Mutations in the orienter loop and in the partitioning loop both result in reduced DNA-binding affinity, reduced polymerase activity, increased point mutability and increased extended mutability [20], [26], [27]. yA692T, equivalent to hA889, is part of a β-sheet which surrounds the catalytic residues in the palm [12]. Mutations in this β-sheet are predicted to reduce polymerase activity [69], and this effect was observed for A692T mutation in yeast [26]. yR467, equivalent to hR574, is part of a subdomain responsible for the intrinsic processivity of Pol gamma [12]. As a matter of fact, mutation yR467W results in a decreased processivity [27].

We showed that these recessive mutations with reduced polymerase activity or processivity are insensitive to antioxidants treatment. Thus, we speculate that different Pol gamma mutations exert their negative effect according to two distinct ways. The first one, rescued by the treatment with antioxidants, can be related to the increased incorporation of 8-oxo-dG: in presence of antioxidant molecules the concentration of oxidized nucleotides and, as a consequence, their incorporation in the mtDNA is reduced. The inability of Pol zeta to rescue these Mip1 mutations may depend partially on the low efficiency (approximately 12%) of Pol zeta to incorporate nucleosides opposite to 8-oxo-dG [70], [71]. The second way, rescued by Rev3 overexpression, could be related to reduced polymerase activity, processivity and/or TLS synthesis of mutated mtDNA polymerase as in the case of C261R, R467W, A692T and G807R mutations. Two modes of action may account for ability of Rev3 to rescue this kind of *mip1* mutations. i) Pol zeta could participate directly to the replication of undamaged mtDNA at the replicative fork partially playing the role of Mip1, as it was recently demonstrated in the case of defective nuclear replisome due to mutations in the replicative polymerases [72]. Interestingly, the rescue is observed for mutations which cause reduced DNA binding affinity (C261R) or for which reduced DNA binding affinity is predicted (R467W and G807R) [27], [69]. ii) Alternately, Pol zeta could partially substitute mutant Mip1 defective of TLS, either during the incorporation opposite to a lesion or the extension from mismatched terminally nucleotides. Reports of translesion synthesis by Pol gamma are limited. I*n vitro* studies showed that Pol gamma from higher eukaryotes is an enzyme with a low TLS activity: it can bypass 8-oxo-dG incorporating dCMP (∼73%) or dAMP (∼27%), but stalls the majority of the time opposite abasic sites and opposite adducts containing benzo[a]pyrene and benzo[c]phenantrene [73], [74]. Moreover, it has been recently reported that human Pol gamma barely bypasses cyclobutane T-T dimers incorporating dAMP (∼80%) or dCTP (∼20%), whereas it extends very inefficiently past the dimer [75]. These observations suggest that DNA polymerase gamma can promote mutagenesis during TLS.

It is worthwhile mentioning that the Rev3 rescue was observed only in the absence of wt Mip1. It could be possible that in heteroallelic strains wt Mip1 out-competes even a 10-fold overexpression of Pol zeta for the stalled replication that is caused by mutant Mip1. As a consequence, putative beneficial effects of overexpression of Pol zeta in heteroallelic strain could be hidden by the presence of wt Mip1.

It has been demonstrated previously that mtDNA instability caused by specific mutations in *MIP1* is reduced by increasing the dNTPs in the cell, either by overexpressing the *RNR1* gene, which encodes the large subunit of the ribonucleotide reductase, or by deleting the *SML1* gene, which encodes an inhibitor of the latter activity [55]. Increased concentration of dNTPs is beneficial since the basal levels of mitochondrial dNTP pools are a limiting factor to maintain mtDNA copy number in yeast [76], [77].

Here we describe that overexpression of Rev3 and increased activity of ribonucleotide reductase have a synergistic effect on reducing the frequency of *petites*. The synergistic effect is probably due to the fact that the activity of Pol zeta but also of Mip1 have improved with the increase of the dNTP pools, which are the substrate of both DNA polymerases. In addition, the *REV3* overexpressing *sml1Δ* strains harboring *mip1* mutations A692T and G807R reached *petite* frequency values which are slightly higher or similar, respectively, to those of wt strain, thus indicating that through genetic modifications it is possible to erase completely the detrimental effects of such *mip1* mutations on extended mutability.

It is worth mentioning that the human Pol zeta protein does not localize to the mitochondria [9]. From the data here described, we suggest that in mammalian cells carrying mutant Pol gamma a beneficial effect could be obtained by overexpressing the yeast Rev3 mutant isoform unable to enter the nucleus but capable of rescuing mtDNA mutability, or as an alternative a mitochondrial localizing version of human Rev3. It has been reported that expression of a yeast mitochondrial repair/recombination enzyme, Cce1, the cruciform-cutting endonuclease, in human cells harboring partially duplicated mtDNA induced the appearance of wt mtDNA, thus demonstrating that expression of a yeast protein could be beneficial for mtDNA stability in mammalian cells [78]. Such a genetic rescue could be associated to a treatment with dNTPs, which seems to be beneficial for the detrimental effects caused by specific mutations in human *POLG*: the processivity defect of human DNA polymerase gamma harboring Y955C mutation was overcome by increasing the dATP or dTTP concentration during *in vitro* synthesis of mtDNA fragments [65] and the treatment with dAMP and dGMP leads to a slight increase in the levels of mtDNA in myoblasts deriving from an Alpers' patient harboring mutations A467T and K1191N in *POLG* [79].
